# Ascaris lumbricoides roundworms visible on a plain -non-contrast- abdominal x-ray in a patient presenting with gastric outlet obstruction

**DOI:** 10.11604/pamj.2017.26.184.12232

**Published:** 2017-03-29

**Authors:** Lykourgos Christos Alexakis

**Affiliations:** 1 Médecins Sans Frontières, Athens, Greece; 2 1^st^ Department of Internal Medicine “G, Gennimatas” General Hospital, Athens, Greece

**Keywords:** Ascariasis, gastric outlet obstruction, radiology

## Image in medicine

A 29 years old male from Bangladesh presented in the emergency department with severe abdominal pain and symptoms of gastric outlet obstruction. Epigastric tenderness, tachycardia, low grade fever, leucocytosis (WBC: 14900/microL) with neutrophilia (Neu:10900/microL) and eosinophilia (Eos:600/microL) were noted, as well as features of obstructive jaundice (increased AST, ALT, and total bilirubin). Stomach dilation was noted on a chest X-ray (A), while in a plain abdominal erect X-ray (C) tubular curvilinear soft tissue densities in a jejunal loop were identified (D). An abdominal CT scan showed stomach dilation and gave the impression of thickening of the pyloric wall and superior part of duodenum (B). In addition, a distended gallbladder, a common bile duct of one cm in diameter and borderline distention of intrahepatic ducts were observed. Subsequently, a nasogastric tube was placed which drained 2 litres of food containing liquid. Antibiotics, opioid analgesics and intravenous fluids were also given. During gastroduodenoscopy a bundle of several entangled Ascaris Lumbricoides roundworms was identified obstructing the duodenum lumen and some of them were removed endoscopically. Following albendazole treatment for three days and passing several dead worms per rectum, the patient recovered and radiological findings resolved completely.

**Figure 1:**
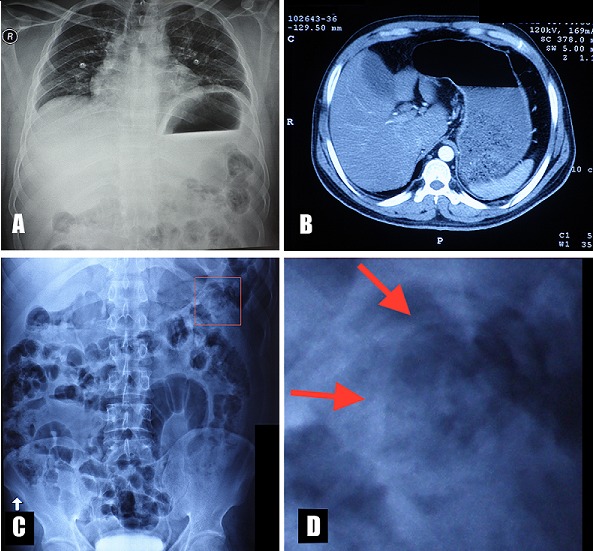
A) chest X-ray showing stomach dilation, prominent gastric air bubble and gastric air-fluid level; B) abdominal CT showing stomach dilation; C) erect abdominal plain X-ray with visible worms in the left upper quadrant (red square); D) detail of panel C (red square) showing tubular or cord-like curvilinear soft tissue densities in a jejunal loop characteristic of ascaris worms (red arrows)

